# Dielectric-Optical Switches: Photoluminescent, EPR, and Magnetic Studies on Organic–Inorganic Hybrid (azetidinium)_2_MnBr_4_

**DOI:** 10.1021/acs.inorgchem.2c00363

**Published:** 2022-03-28

**Authors:** Magdalena Rok, Bartosz Zarychta, Rafał Janicki, Maciej Witwicki, Alina Bieńko, Grażyna Bator

**Affiliations:** † Faculty of Chemistry, University of Wroclaw, 14 F. Joliot-Curie, 50-383 Wroclaw, Poland; ‡ Faculty of Chemistry, University of Opole, 45052 Opole, Poland

## Abstract

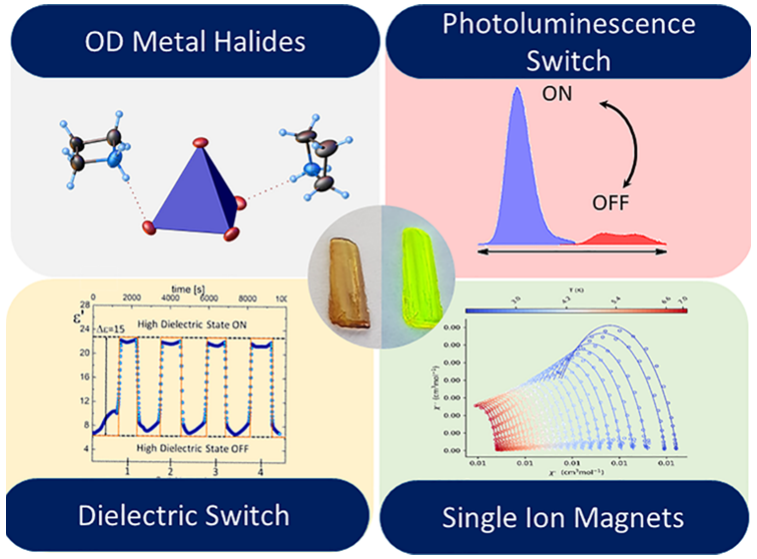

A new organic–inorganic hybrid, **AZEMnBr**, has been synthesized and characterized. The thermal differential scanning calorimetry, differential thermal analysis, and thermogravimetric analyses indicate one structural phase transition (PT) at 346 and 349 K, on cooling and heating, respectively. **AZEMnBr** crystallizes at 365 K in the orthorhombic, *Pnma*, structure, which transforms to monoclinic *P*2_1_/*n* at 200 K. Due to the X-ray diffraction studies, the anionic MnBr_4_^2–^ moiety is discrete. The azetidinium cations show dynamical disorder in the high-temperature phase. In the proposed structural PT, the mechanism is classified as an order–disorder type. The structural changes affect the dielectric response. In this paper, the multiple switches between low- and high- dielectric states are presented. In addition, it was also observed that the crystal possesses a mutation of fluorescent properties between phase ON and OFF in the PT’s point vicinity. We also demonstrate that EPR spectroscopy effectively detects PTs in structurally diverse Mn(II) complexes. **AZEMnBr** compounds show DC magnetic data consistent with the *S* = 5/2 spin system with small zero-field splitting, which was confirmed by EPR measurements and slow magnetic relaxation under the moderate DC magnetic field typical for a single-ion magnet behavior. Given the above, this organic–inorganic hybrid can be considered a rare example of multifunctional materials that exhibit dielectric, optical, and magnetic activity.

## Abstract 2

A new multifunctional optical-dielectric bistable switch (azetidinium)_2_[MnBr_4_] has been synthesized, and a description of its properties (thermal, structural, electric, magnetic, and photoluminescence) is presented. EPR measurements and slow magnetic relaxation under the moderate DC magnetic field indicate the typical SIM behavior.

## 1 Introduction

Since 2009, lead-halide hybrid perovskite materials have become a flashpoint in functional materials because of their photoluminescent (PL)^1,2^ and electroluminescent (EL)^3−5^ properties. Moreover, due to the high quantum efficiency of photoluminescence (PLQY), high absorption coefficient, and high mobility of the charge carriers,^6,7^ halo-Pb perovskites may be introduced into the group of next-generation materials. Currently, solar cells, based on methylammonium lead halide, are promising candidates for the cheap preparation from solution and highly efficient solar cells with short energy payback time. Since the first reports appeared, certified power conversion efficiency has now exceeded 25%^5,8,9^ and even reaching the value of 30%^10^ in monolithic perovskite tandem solar (photovoltaic) cells. Additionally, lead halide perovskites with exceptional optical properties have been used as a phosphor component in the light-emitting diode (LED) applications, promising to replace traditional rare-earth phosphors due to their earth-occurring elements and low-cost synthesis. Unfortunately, despite such a good profit, the high lead toxicity and poor stability limit their use. Therefore, developing highly stable lead-free metal halide materials is crucial for basic scientific research and technological sustainability. Among the environmentally friendly hybrids, the compounds based on Mn(II) appear like a good choice.^11^ This choice is dictated by the variety of properties observed in the systems based on Mn(II). The interest in new manganese-based hybrids results from their multifunctionalities, such as ferroelectricity, piezoelectricity, photoluminescence, and dielectric constant switching.^11−21^ A perfect example of blue-light excited red emission, with a high PL quantum efficiency (PLQY) of 55.9%, is an organic–inorganic hybrid (guanidine)_2_MnCl_4_. In the crystal structure, every three octahedral coordinated [MnCl_6_]^4–^ units share faces forming unique trimeric [Mn_3_Cl_12_]^6–^ linear chains along the *b*-axis.^22^ What is worth emphasizing is that the hybrid was obtained via a facile mechanochemical method. Crystals with A_2_MnX_4_ stoichiometry typically emit green light, but there are unique cases of red emission of the tetrahedral configuration of Mn(II) halides.^23,24^ Such a rarely reported red emission results from the coordination environment between the anions and cations and the distortion degree of the metal framework.

As a result of phase changes in stimuli-responsive materials, the states with different physical or chemical properties are generated. One such example is the ferroelectricity phenomenon observed in hybrids based on Mn(II) halides. In the group of bromide derivatives, a crystal with a pyrrolidinium cation is an excellent example, with an ABX_3_ perovskite-type structure, and a spontaneous polarization is generated with a value equal to 5.2 μC/cm^2^.^25^ In the case of A_2_BX_4_, where A is a diisopropylammonium cation, the recorded polarization (1.2 μC/cm^2^) was noticeably lower than that for the previous compound. However, ferroelectric properties were observed in a broad range of temperatures up to 420 K.^14^ In another inspiring system [(CH_3_)_3_NH]_3_(MnBr_3_)-(MnBr_4_), the ferroelectricity was experimentally proven for the first time in the antiperovskite structure (A_3_BX).^19^, whereas the crystal of trimethylchloromethyl ammonium trichloromanganese(II) [Me_3_NCH_2_Cl]MnCl_3_, (TMCM·MnCl_3_) is one of the first examples of a single-phase organic–inorganic perovskite that exhibits a piezoelectric coefficient d_33_ of 185 pC/N.^21^ In the discussed hybrids, the phase transformations caused the dielectric constant switching between two low and high states. Such switchable materials are of great interest because this property can be used in two ways. In integrated circuits, hybrids can be incorporated, which in the “off” low-dielectric phases form so-called low-*κ*-dielectrics. On the other hand, the highly dielectric “on” phases can be used for energy storage. In hybrids, the most crucial influence on the tunable and switchable properties is the change in the dynamics of the organic part, that is, the dipole reorientation. Therefore, selecting an appropriate rotator (significant dipole moment, small particle size, spherical structure, etc.) is crucial in constructing switchable molecules.

This time we chose the azetidinium (AZE) cation because its hybrid with ZnX_2_ (X = Cl, Br) showed excellent switchable properties.^26^ Both the chloride and bromide analogues of AZE_2_ZnX_4_ crystals underwent one phase transition (PT) in the solid state at 342 and 356 K for X = Cl and Br, respectively. Encouraged by the structural tunability of these crystals and their electrical and optical properties, we obtained a crystal, hitherto not reported in the literature, with the A_2_BX_4_ stoichiometry due to the reaction of MnBr_2_ with azetidine in the presence of aqueous HBr. According to the thermal results, the crystal undergoes one PT, so we conducted a complete structural analysis and measured the dielectric constant switchability and the EPR spectra in a broad temperature range. Because the crystal exhibits PL properties, we have performed optical tests as a function of temperature. The analysis of magnetic parameters and theoretical calculations were complementary to the rest of the results. Combining one material with different physical properties is a very promising approach to creating novel materials with rich functionality.

## 2 Experimental Section

### 2.1 Sample Preparation

#### 2.1.1 **(C**_**3**_**H**_**8**_**N)**_**2**_**[MnBr**_**4**_**]** (**AZEMnBr**)

**Figure 1:**
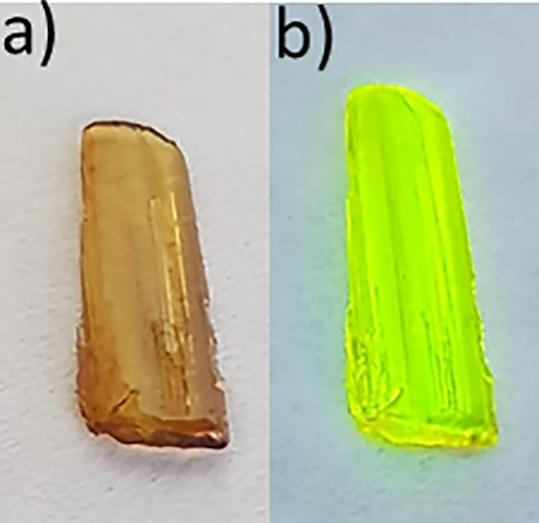
Single crystals of **AZEMnBr** crystallized from aqueous solution (a) under ambient light and (b) under UV light.

4.4 g of MnBr_2_·4H_2_O (Sigma-Aldrich, 98%, 15 mmol) was dissolved in deionized water, and concentrated hydrobromic acid (Sigma-Aldrich, 48%, 4.5 mL, 30 mmol) was added dropwise to the solution. Next, azetidine (Sigma-Aldrich, 98%, 2 mL, 30 mmol) was added in part to the solution placed in the ice bath. By slow evaporation at room temperature, green crystals in the shape of the block were obtained (see Figure 1). The composition of the compound was confirmed by elemental analysis to be C: 14.5% (theor. 14.68%), N: 5.69% (theor. 5.71), and H 3.16% (theor. 3.29). Powder X-ray diffraction (XRD) verified the phase purity (see Figure S1, Supporting Information). XRD was recorded in the range 2θ = 5–80° with the step 2θ = 0.024° and 1s counting time using a D8 ADVANCE X-ray diffractometer from Bruker. The Ni-filtered Cu *K*_α1_ radiation (λ = 1.540596 Å) from a Cu X-ray tube was applied. The structure factors from the single crystal XRD experiment at 200 K were used for the phase identification. The pattern has been calculated and then refined using the Rietveld approach implemented in Maud software, ver. 2.992.^27^

### 2.2 Thermal Analysis

Thermal differential scanning calorimetry (DSC) measurements were carried out under the following conditions: the temperature range was from 260 to 380 K with a cooling and heating rate of 10 K/min., and the instrument used was Metler Toledo DSC 3. Thermogravimetric measurements (TGA/DSC) were performed on a TGA-DSC3 + instrument in the temperature range from 290 to 900 K, with a heating rate of 5 K·min.^–1^. Scanning was carried out in flowing nitrogen (flow rate: 1 dm^3^·h^–1^).

### 2.3 X-ray Crystallographic Studies

X-ray measurements of the **AZEMnBr** were performed on a CCD Xcalibur diffractometer (graphite-monochromated Mo *K*_α_radiation, *λ* = 0.71073 Å) at 200 (phase **II**) and 365 K (phase **I**). For all data, Lorentz and polarization corrections were applied to the reflection.^28^ The SHELX program package^29^ was used to solve the structures by direct methods. Graphics were made with Mercury 2020.1.^30^ The positions of the hydrogen atoms were refined using a riding model with constrained temperature parameters. All non-hydrogen atoms were located from difference-Fourier electron-density maps. The experimental conditions and XRD data are given in Table S1 (Supporting Information). The coordinates of atoms and other parameters for structures were deposited with the Cambridge Crystallographic Data Centre [no. 2069243 (200 K) and 2069244 (365 K).

### 2.4 Electric Properties

The complex electric permittivity measurements were performed by using an Agilent E4980A LCR meter. Polycrystalline samples were pressed in pellets with following geometrical parameters: *S* = 20 mm^2^ and *d* = 0.78 mm. The dielectric response was measured in the temperature range from 200 to 360 K and the frequency range from 135 Hz to 2 MHz. The measurement was performed under a nitrogen atmosphere.

### 2.5 Absorption and Luminescence Spectra

The absorption spectra of monocrystals were measured on a Cary 5000 spectrophotometer. The temperature measurements of the crystals were performed as follows: the sample was placed into a small teflon holder, which was fixed in a 1 cm quartz cuvette filled with paraffin oil. The temperature of the samples was regulated using a temperature controller TC 125.

The experimental oscillator strengths (*P*_exp_) were determined by using eq 1
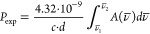
1where *c* is the concentration of the Mn(II) ion in M, *d* is the length of the optical way in cm, and *A*(ν̅) is the absorbance as a function of the wavenumber in cm^–1^. The luminescence decay curves of crystals were detected on an Edinburgh Instruments FLS 920 spectrometer with the monitored emission at 530 nm.

### 2.6 EPR Measurements

EPR experiments were carried out for powder samples using a Bruker Elexsys E500 spectrometer operating at ∼9.5 GHz (X-band) frequency. The spectrometer was equipped with an NMR teslameter and a frequency counter. The temperature was controlled by using a Bruker ER 4131VT variable temperature accessory and stabilized for 15 min before a spectrum was recorded. We set the amplitude and frequency of the modulating field to 5 G and 100 kHz, respectively and the microwave power to 10 mW. The spectra were simulated using a pure Lorentzian line shape. The g factors, linewidths (Γ), and relative weights of Mn(II) centers were determined from the numerical simulations. The linewidths we report are the full width at half height. They are related to the distance between the inflection points (Γ_PP_) via Γ_PP_ = Γ/√3. The EPR spectra were simulated using EasySpin 5.2.30.^31,32^

### 2.7 Theoretical Computations

Theoretical calculations were conducted using ORCA 4.2.1 software.^33,34^ In all the calculations, scalar relativistic effects were included using the zeroth-order regular approximation (ZORA) following the model potential approximation proposed by van Wüllen.^35^ The respective ZORA-def2-TZVP basis set was employed for all atoms.^36^ To speed up the calculations, the resolution of identity approximation was used.^37^ The auxiliary basis set was generated using the AutoAux procedure.^38^ In the calculations, the structures determined from the XRD experiments were used, but the positions of hydrogen atoms were optimized using the functional B3LYP.^39−42^ The state-averaged complete active space self-consisted field (CASSCF)^43−45^ in concert with strongly contracted N-electron valence perturbation theory to second order (NEVPT2)^46−48^ was used to calculate the zero-field splitting (ZFS) parameters D and E. All states were equally weighted in these calculations, and quasi-degenerate perturbation theory was used.^49,50^ The B3LYP quasi-restricted orbitals^51^ were the initial guess for the CASSCF calculations. Using the coupled perturbed method, the g tensor was calculated at the B3LYP and PBE0^52^ theory level.^53,54^

### 2.8 Magnetic Measurements

The DC magnetic measurements in the temperature range 1.8–300 K (*B*_DC_ = 0.1 T) and variable—field (0–5 T) (at low temperature) were taken using the Quantum Design SQUID magnetometer (MPMSXL-5- type) with ca 27 mg of the sample. Corrections were based on subtracting the sample—holder signal and contribution χ_D_ estimated from the Pascal constants.^55^ No remnant magnetization has been detected. Variable-temperature (2–7 K) alternating current (AC) magnetic susceptibility data were taken with same apparatus and samples using *B*_AC_ = 0.3 mT amplitude of the oscillating field. To prevent any displacement of the sample due to magnetic anisotropy, magnetic measurements were performed by crushing the crystals and restraining them.

## 3 Results and Discussion

### 3.1 Crystal Structure Determination

**Figure 2:**
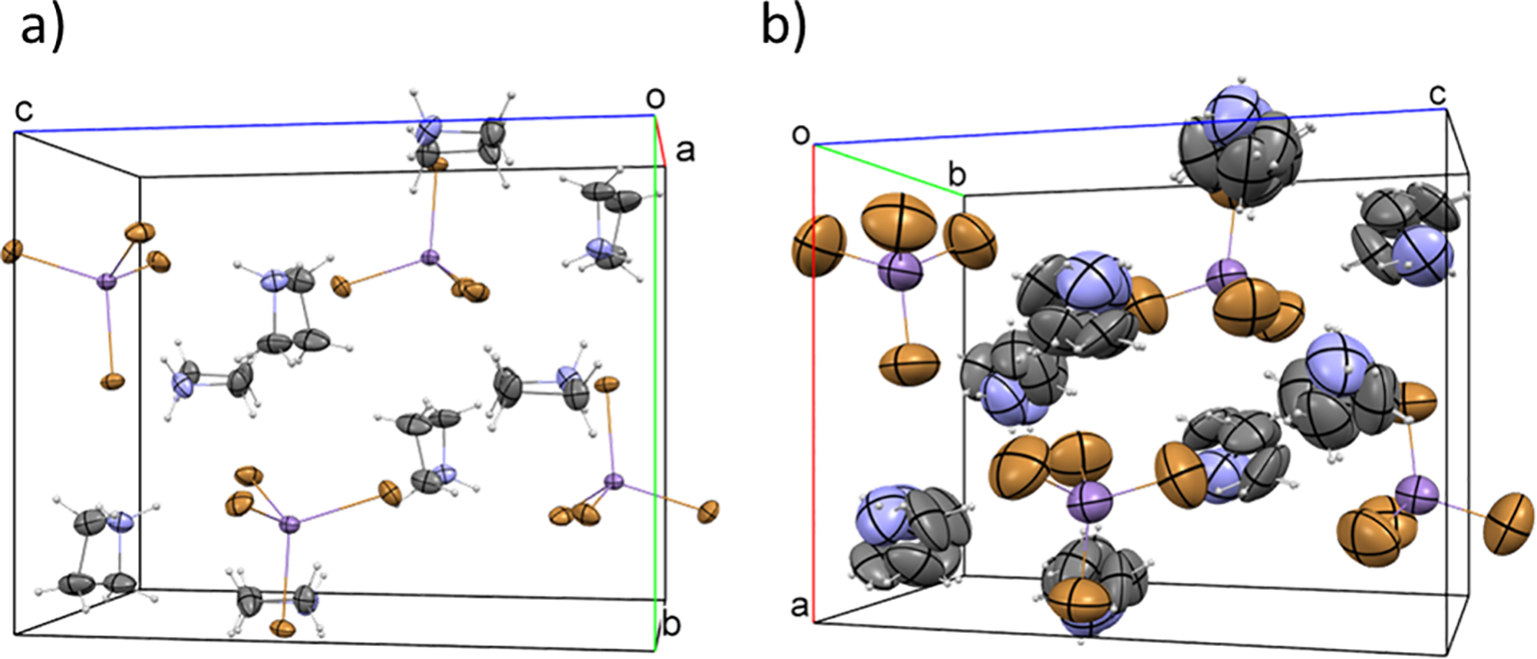
Packing of the **AZEMnBr** structure at (a) 200 K (phase **II**) and (b) 365 K (phase **I**). Displacement ellipsoids are plotted at the 50% probability level.

The crystal packings of **AZEMnBr** at 200 and 365 K are depicted in Figure 2. Geometry parameters of hydrogen bonds are summarized in the Supporting Information (Tables S2 and S3). The structure is composed of MnBr_4_^2–^ tetrahedra and two C_3_H_8_N^+^ cations. At 365 K, the geometrical parameters of the cations (Table S2, Supporting Information) are unreasonable due to the complex character of the disorder; for this reason, the geometry of the organic part will not be further discussed. On lowering the temperature, the blocking of the rotation of cations takes place. In phase (**II**), the cation motions are frozen, while the molecules are disordered in phase (I).

#### 3.1.1 Structure at 200 K, Phase II

Phase **II** is monoclinic (space group *P*2_1_/*n*). The asymmetric part of the unit cell consists of one [MnBr_4_]^2–^ moiety and two protonated AZE cations. The crystal is isostructural with the (C_3_H_8_N)_2_[ZnCl_4_] crystal structure reported by Shi et al.^26^ The tetrahedral coordination of Mn^2+^ is distorted. The Mn–Br valence bonds differ by 0.034 (6) Å (Table S2, Supporting Information). The Br–Mn–Br angles range from 104.56 (2)° to 113.57 (2)°. The AZE cations’ geometry is normal. The molecules are slightly folded as the deviation from the mean plane of plane-fitted atoms (N1, C2, C3, and C4 and N5, C6, C7, and C8) amounts to 0.0309 and 0.0931 Å for N1 and N5 cations, respectively. The crystal structure is stabilized by a compound hydrogen-bonding network (Table S3, Supporting Information). Every bromide ligand is connected to at least one rather strong N–H···Br hydrogen bond forming a three-dimensional structure. The geometry of HBonds does not influence the structure of the [MnBr_4_]^2–^ anion; however, its number does. Bromide atoms, which are involved in two hydrogen bonds, form the longest Mn–Br bonds (Mn1–Br1 and Mn1–Br3′), while two remaining Br atoms, which form shorter valence bonds, are linked to cations by single H bonds.

#### 3.1.2 Structure at 365 K, Phase I

**Figure 3:**
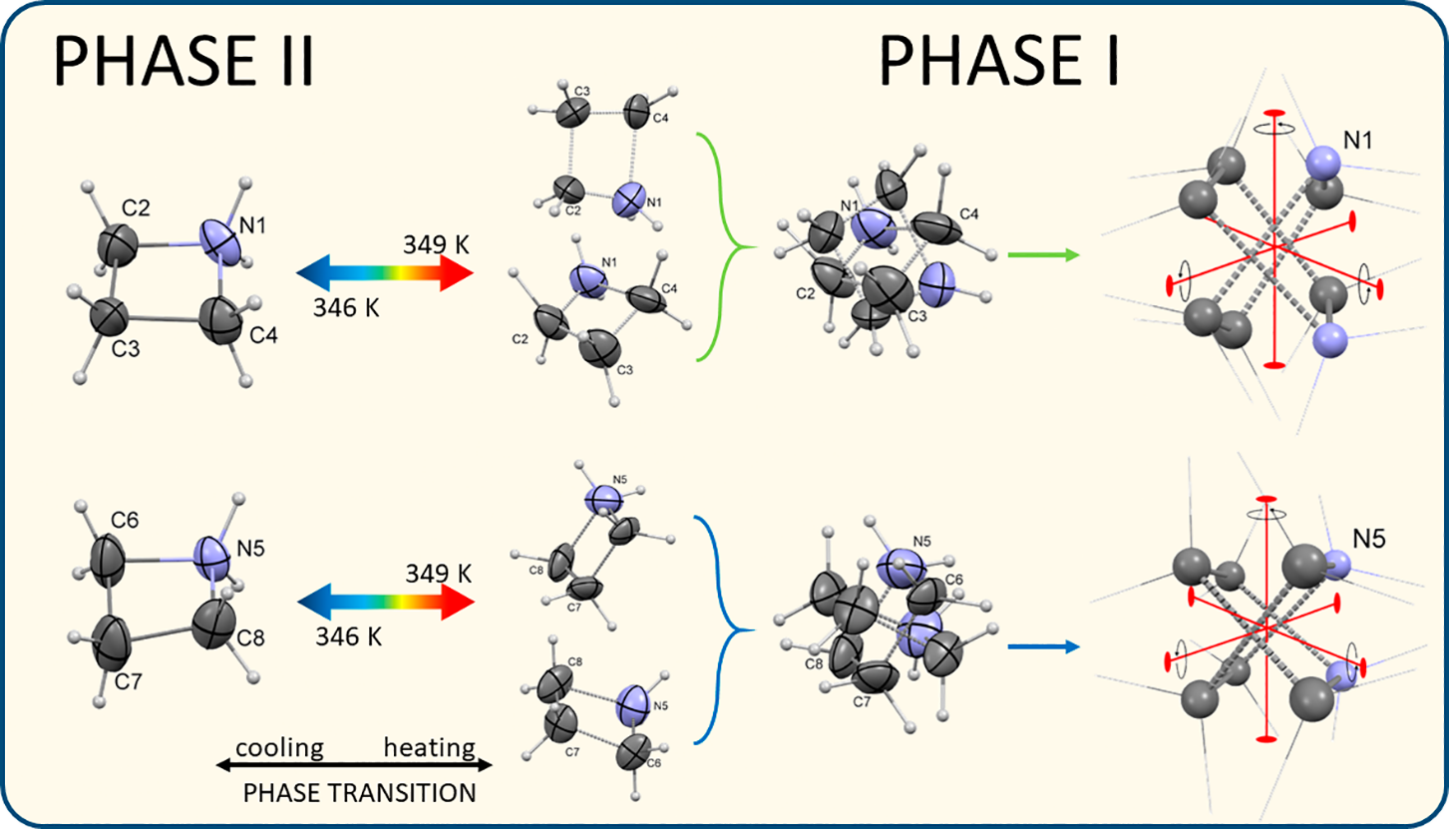
Distortion models for N1 and N5 cations at 200 (phase **II**) and 365 K (phase **I**). Displacement ellipsoids are plotted at the 50 and 10% probability level for phase **II** and **I**, respectively. At phase **I**, the orientation of pseudo-2-fold axes (pseudo *D*_4h_ symmetry) generates overall disorder of the N1 and N5 cations.

The structure of **AZEMnBr** at 345 K is orthorhombic (*Pnma*). The transition stimulates major symmetry change, that is, the crystallographic symmetry elements extend from (*E*, *C*_2_, σ_h_, *i*) in the low-temperature phase to (*E*, *C*_2_, *C*_2_^′^, *C*_2_″, *i*, σ_h_, σ_v_, σ_v_^′^) above-*T*_c_ temperature. The main difference in phase (II) is related to the AZE cation dynamics. At first glance, at 365 K, both cations (N1 and N5) are disordered over two sites (Figure 3). Nevertheless, as shown in the figure, each atom of C and N may occupy all other positions in the disorder model. This observation is justified by at least three premises: (i) The model is relatively symmetrical in accordance with a pseudo-*D*_4h_ symmetric site. (ii) The hydrogen bond pattern suggests the multi-positional occupation of the nitrogen atom in the model. (iii) The previous study on (C_3_H_8_N)_2_[ZnCl_4_] showed in the isostructural HT phase a highly orientational disorder of the [A]_*m*_[B]_*n*_ (A = spherical-like cation, B = tetrahedral anion) type.^26^ As suggested by the authors, the disorder displays a ball-like model, which has been already well studied.^56−58^ Unfortunately, the authors failed to refine the model of the disorder, introducing a single atom as a representation of the whole cation in the crystal structure.

The [MnBr_4_]^2–^ moiety is not affected by the temperature change. However, the arrangement of the bromide ligands around the central Mn^2+^ ion at 365 K deviates less from the ideal tetrahedron than that in the 200 K phase. The Mn–Br bond lengths differ by only 0.02(2) Å, while the Br–Mn–Br angles range from 106.63(6)° to 112.94(6)°. The tetrahedral coordination distortion can be easily estimated by the deviation parameters for bond lengths and valence angles, that is, Δ and σ, respectively
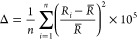
2
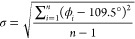
3where for eq 2, *R̅* is the average Mn–Br bond length and *R*_i_ the individual Mn–Br distance (*n* = 4), and for eq 3, ϕ_*i*_ is the individual Br–Mn–Br valance angle (*n* = 6). The Δ parameter amounts to 0.97 for the structure at 365 K, while at 200 K, the tetrahedron is more distorted as Δ amounts here to 2.88. The same trend is observed for valance angles, where the values of σ amount to 3.01 and 6.05 for the structure at 365 and 200, respectively.

### 3.2 Phase Transition Screening

**Figure 4:**
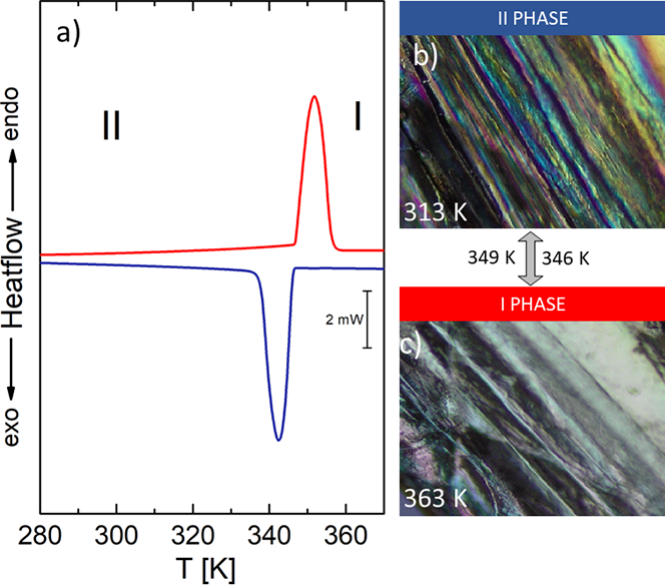
(a) DSC runs measured for **AZEMnBr** (solid line) upon heating (red) and cooling (blue) run. The observation of the crystal under the polarized microscope at (b) 313 K (phase **II**) and (c) 363 K (phase **I**).

The DSC measurements first checked the thermal-triggered PT in the compound **AZEMnBr** (Figure 4a). Throughout the measured temperature range of 280–370 K, the compound exhibits reversible endo- and exothermic peaks at 349 and 346 K on heating and cooling, respectively. For clarity, below 349 K, the low-temperature phase is entitled as **II** and the high-temperature phase as **I**. According to the data in Table S4 (Supporting Information), the corresponding entropy change (Δ*S*) in the PT, calculated from the enthalpy change measured, is equal to 34.6 J/mol·K (for the heating cycle). According to the Boltzmann equation, Δ*S* = *R* ln *N*^2^, where *R* is the gas constant, and *N* is the proportion of the numbers of the corresponding distinguishable geometric orientations allowed in phases **I** and **II**. The power coefficient of 2 relates to two cation molecules in 1 mol of the compound. The value of *N*, calculated from the Δ*S* value, is about eight, which indicates that the PT is of the order–disorder type. In addition to the PT in the solid state, the transition from solid to liquid decomposition is observed at 442 K (see TGA/DSC Figure S2, Supporting Information).

According to the crystal structure analysis of **AZEMnB**r, the crystallographic system changes during the PT. This is the first necessary condition to classify the PT to the ferroelastic–paraelastic type. In this case, the transition is from the ferroelastic-monoclinic space group (phase II) with four symmetry elements to orthorhombic-paraelastic (phase **I**), where we have eight elements of symmetry. It means that two different types of ferroelastic domains should be observed in phase **II** under a polarizing microscope. Moreover, according to Aizu’s classification, the transition should be defined as *mmm*F2/*m* (#62) as a full ferroelastic–paraelastic PT. Due to the poor quality of the crystal, no sharp domain boundaries are visible in the images taken with the polarized microscope (see Figure 4b,c).

### 3.3. Electric Properties

**Figure 5:**
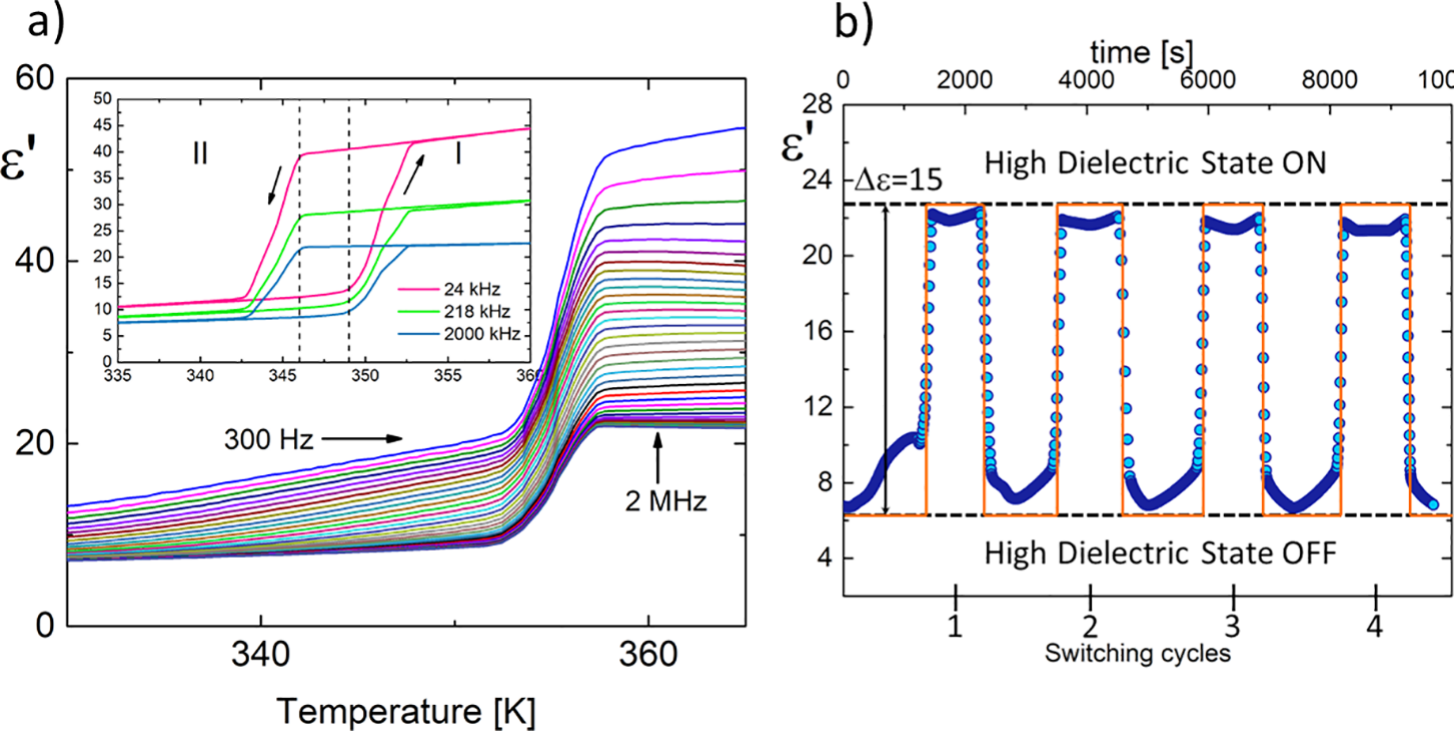
(a) Temperature dependence of the real (ε′) part of the complex dielectric constant (ε*) measured for **AZEMnBr**. The inset presents the heating and cooling cycles for *f* = 24 kHz, 218 kHz, and 2 MHz. (b) Cycles of switching ON and OFF of ε′ between 300 and 370 K measured at 2 MHz.

Thermally activated molecular rotations and structural changes strongly influence the dielectric response around PT. The response changes are all the more spectacular when they concern a component endowed with a permanent dipole moment. In the case of **AZEMnBr**, the phase change is primarily related to the AZE cation reordering (AZE^+^). However, according to the structural analysis, the contribution of the anionic part [MnBr_4_]^2–^ cannot be neglected either. Consequently, we observe the transition between two states: low (OFF) and high (ON) dielectric. The order-to-disorder transition results in a switch of the dielectric constant between these two states. The measurements of the dielectric constant confirm this on powdered samples in the frequency and temperature range of 500 Hz–2 MHz and 300–370 K, respectively (Figure 5a). The graphs show the dielectric transition between OFF and ON states at 349 K (heating cycle), consistent with the DSC results corresponding to the structural PT.

Notably, the dielectric response around PT indicates a sharp switching property (Figure 5b). Based on phase **II** structural analysis, two AZE^+^ cations and the anion are completely ordered in the structure. At this state, no dielectrically active dipolar reorientation movements are observed. Therefore, the values of ε′ contain only contributions from electron and ion shifts and remain in a state with a low dielectric constant (ε′ ∼7 for 2 MHz at 330 K). In the vicinity of PT, each AZE^+^ cation becomes completely disordered, contributing to the dipole reorientation by increasing the dielectric constant value (ε′ ∼ 23 for 2 MHz at 360 K).

Multiple switching between low- and high-dielectric states is a desirable feature for applications such as smart electronics, switches, sensors, and transistors. Figure 5b shows an example of reversible dielectric switching between “ON” and “OFF” states at 2 MHz and illustrates the results obtained from several consecutive measurement cycles performed on a polycrystalline sample. Before the transition, the dielectric constant value for all crystals is about seven. After PT, a dramatic jump ε′ to 22 was observed. In the case of **AZEMnBr**, no weakening of the dielectric signal was observed during cyclic processes, proving the high thermal and electrical stability of the samples. The increment (Δε) and the ratio of the dielectric switching (ε_ON_/ε_OFF_) measured at 2 MHz equal 15 and 3 ± 0.3, respectively.

### 3.4 Absorption and Luminescence Properties

**Figure 6:**
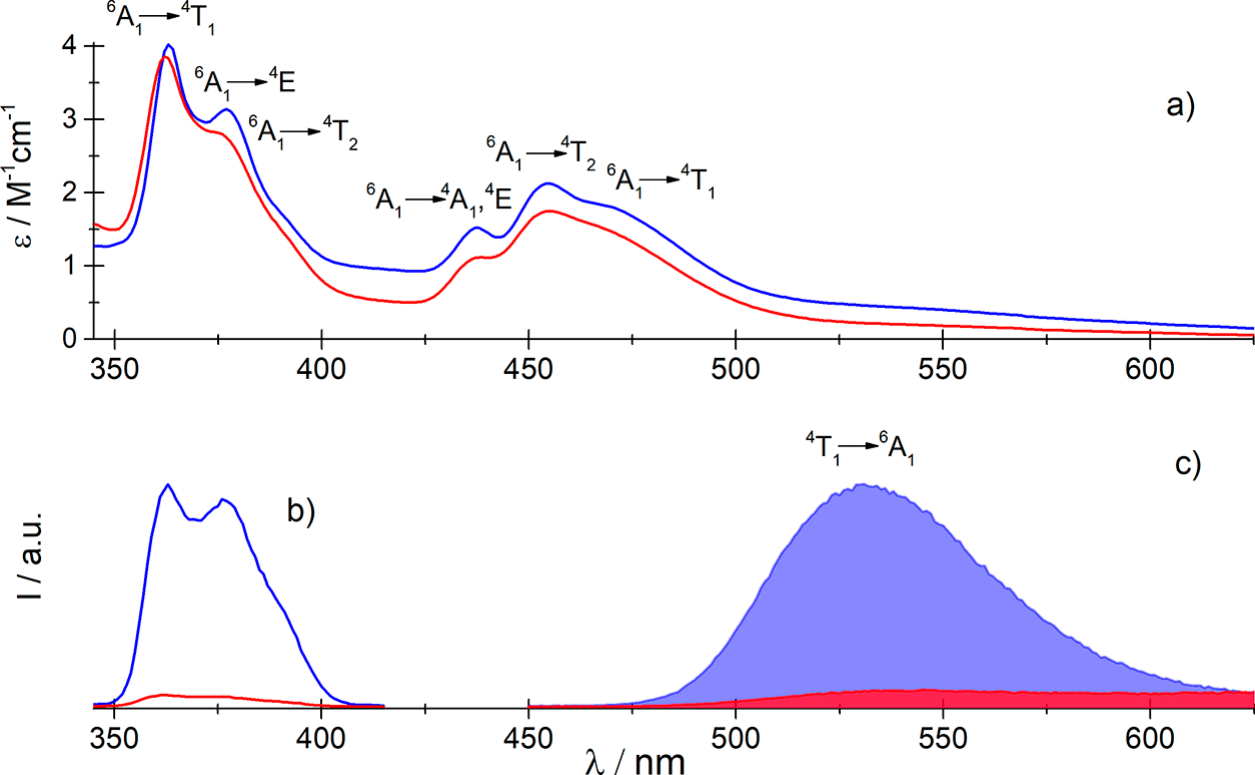
UV–Vis spectra: (a) absorption, (b) excitation luminescence, and (c) emission of the **AZEMnBr** monocrystals.

To elucidate how the PT is reflected in the electronic structure of the molecular anion [MnBr_4_]^2-^, the UV–Vis absorption and luminescence spectra of the compound under study were measured. In the UV–Vis absorption spectrum between 24 000 and 30 000 cm^–1^, weak bands attributed to the intraconfiguration 3d–3d transitions are observed (Figure 6).

The spectral pattern of the bands is characteristic of the Mn(II) systems, which possess tetrahedral symmetry. Two distinct groups of bands centered at ∼22 000 (hereinafter referred to as A) and ∼27 100 cm^–1^ (hereinafter referred to as B) are assigned to the spin-forbidden transitions between ground ^6^A_1_ and quartet states. The energy of the individual states is presented in Table 1.

**Table 1:** Energy of Electronic Transitions in UV–Vis Absorption and Luminescence Spectra and Oscillator Strength of the Group of Bands A and B

		Δ*E*/cm^–1^		P × 10^8^	
	transition	293 K	363 K	293 K	363 K
band A	^6^A_1_ → ^4^T_1_	∼21 290	∼21 290		
	^6^A_1_ → ^4^T_2_	21 980	21 980	1260	1190
	^6^A_1_ → ^4^A_1_,^4^E	22 850	22 800		
band B	^6^A_1_ → ^4^T_2_	∼25 640	∼25 640		
	^6^A_1_ → ^4^E	26 530	26 600	1980	2020
	^6^A_1_ → ^4^T_1_	27 530	27 590		

As seen, the energy of the band maxima does not change with the increase in the temperature. The derived spectroscopic parameters Racah (*B* = 690 cm^–1^) and crystal field splitting (Δ = 240 cm^–1^) are very similar to those reported for the other tetrahedral Mn(II) bromide systems.^59^ There are only minute changes in the energy of the band maxima. However, the relatively well-separated band ascribed to the ^6^A_1_ → ^4^A_1_,^4^E transition is bathochromically shifted by 50 cm^–1^ due to the nephelaxetic effect. This transition is particularly sensitive to the covalence effect as its energy depends only on B and C Racah parameters.^60^ The shortening of the Mn^2+^-Br^–^ bond lengths (by about 0.05 Å) in the high-temperature phase (**I**) increases the covalency, and the energy of the ^6^A_1_ → ^4^A_1_,^4^E band decreases. The intensity of bands A and B slightly depends on the temperature changes, and thus, the oscillator strength changes no more than a few percent.

The excitation and emission spectra were also measured at different temperatures (Figure 6). The shape and the energy of band A in the excitation luminescence spectra are similar to those observed in the absorption spectrum. This result may suggest that the structure of the [MnBr_4_]^2–^ anion is similar in both ground and excited electronic states. In the emission spectra recorded at different temperatures between 293 and 343 K, a strong band centered at 18 750 cm^–1^ is observed and attributed to the ^4^T_1_ → ^6^A_1_ transition. The Stokes shift of this band is about 2540 cm^–1^. Above 343 K, the luminescence is strongly quenched, and simultaneously, the other weak band centered at 15 620 cm^–1^ appears in the spectrum. Interestingly, the red emission is characteristic of octahedral [MnBr_6_]^3–^ systems, although in the studied compound, there are only monomeric, tetrahedral [MnBr_4_]^2–^ units, which are well separated from each other.

**Figure 7:**
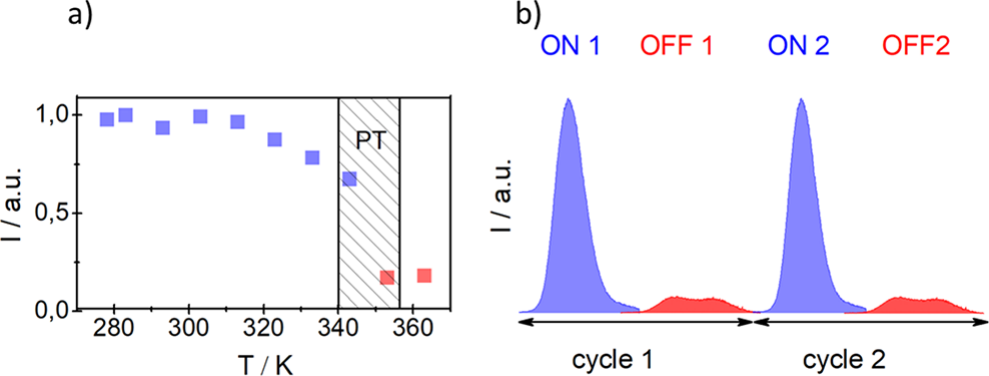
(a) Dependency of normalized emission intensity versus temperature. (b) Cycles of switching ON and OFF of emission spectra at 313 K (phase **II**) and 363 K (phase **I**) for an excitation wavelength of 530 nm.

The temperature-dependent changes in the integral intensity of the emission band are reversible. Figure 7 presents the optical switchable properties of the compound **AZEMnBr**, where variable temperature emission spectra were measured in the range between 313 K (phase **II**) and 363 K (phase **I**). The spectra below the PT show a higher emission peak than the intensity after the PT point. In phase **I**, the intensity of the emission peak becomes four times smaller than that observed for low-temperature phase **II**. During the subsequent cooling cycle from phase **I** to **II**, the spectrum exhibits an emission peak at the same energy, revealing the reversible switching of the fluorescence intensity. It means that **AZEMnBr** possesses additional switchable properties; it not only has a dielectric mutation but also a mutation of fluorescent properties between the states ON and OFF in the PT’s point vicinity. The switching ratio in this case equals *I*_ON_/*I*_OFF_ = 4 ± 0.5. This is the next example of the material, in which a dielectric and fluorescent double switch may be used in the intelligent material application.^61^ Additionally, it was found that the luminescence lifetime is about two times shorter above 353 K. The rapid decrease in the luminescence intensity and lifetime, caused by high-temperature PT, has not been reported in the literature so far (see Figure S3, Supporting Information).

### 3.5. EPR Spectroscopy

**Figure 8:**
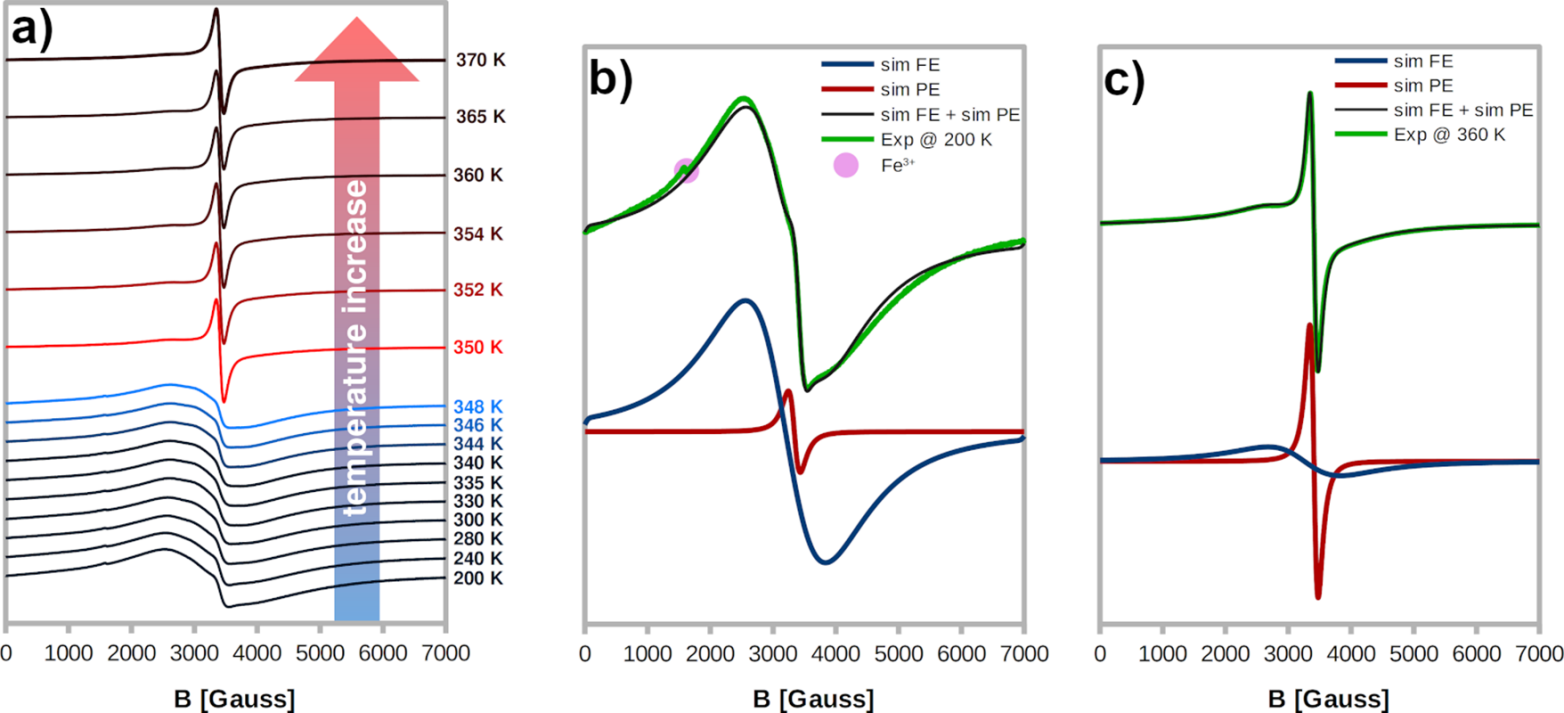
Temperature-dependent EPR spectra of Mn(II) ions in **AZEMnBr** (a) and simulations of the spectra recorded at 200 (b) and 360 K (c).

**Figure 9:**
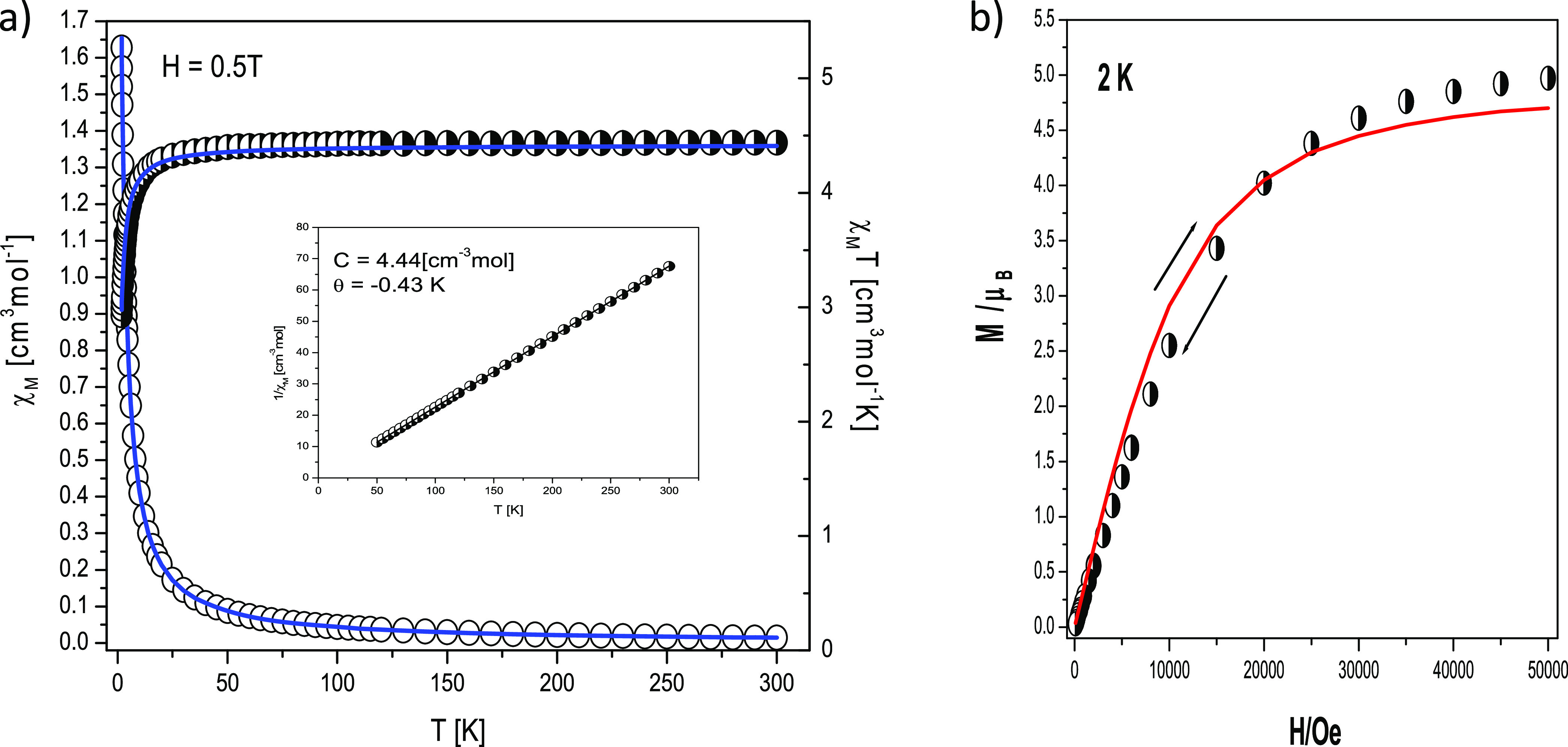
DC magnetic data for **AZEMnBr**. (a) Thermal dependencies of *χ*_M_*T* (half -open circles) and *χ*_M_ (open circles); the insets show thermal dependencies of inverse magnetic susceptibility; and (b)—field dependence of the magnetization per formula unit. The solid lines (on both graphs) are calculated using the spin Hamiltonian given in eq 4.

EPR spectroscopy was proved as an effective tool to detect PTs in structurally diversified Mn(II) complexes.^62−67^ Temperature dependence of the X-band EPR spectra for crystalline powder samples of **AZEMnBr** was monitored in the 200–370 K, with close attention near the structural PTs (Figure 8). At 200 K (in ferroelastic phase **II**), the spectrum consists of a single, very broad, and unresolved line. A closer inspection revealed that this line is the superposition of two signals, henceforth labeled as ferroelastic (FE) and paraelastic (PE), due to structurally different Mn(II) centers. This spectrum was successfully simulated, as shown in Figure 9b, assuming *g* = 2.014 and Γ = 0.23 T for FE, *g* = 2.005 and Γ = 0.024 T for PE, and relative weights 0.99 and 0.01 for FE and PE, respectively. The broadening of the EPR lines prevented the observation of the hyperfine splitting due to ^55^Mn.

Strong dipole–dipole interactions between the concentrated paramagnetic Mn(II) ions can explain the very broad line of the FE signal, which is dominant at 200 K. The shape of this line at its both ends indicates the averaged signals of the low-intensity ZFS transitions *M*_S_ = ±3/2↔ ±5/2 and *M*_S_ = ±1/2↔ ±3/2. This is not uncommon for powder Mn(II) spectra and suggests that |*D*| is comparable with the microwave quantum energy (9.5 GHz–0.3 cm^–1^).^68^ Such a magnitude of |*D*| stays in line with |*D*| = 0.2 cm^–1^ determined by the fitting of magnetic susceptibility (discussed below). To confirm this magnitude of *D*, we carried out the CASSCF/NEVPT2 computations with the active space of 29 electrons in 17 orbitals [the inclusion of all 3d Mn(II) and 4p bromine orbitals], that is, CAS(29,17), and performed calculations for 37 sextet, 24 quartet, and 75 doublet states (detailed discussion given in the Supporting Information as Figure S4 and Table S5). These computations showed that the *D* parameter value for **AZEMnBr** is small, and its sign is positive (*D* = +0.11 cm^–1^).

As the temperature of the powder sample **AZEMnBr** was raised, the EPR spectrum initially remained unaltered, but above the point of PT (at 350 K), its shape changed markedly, showing that the Mn(II) ions are sensitive to the transition and that the observed ferroelastic–paraelastic PT is of the first order (Figure 8a). The narrower line, labeled as PE, became the dominant signal, while the g parameters and linewidths for FE and PE were only slightly affected. The high- and low-temperature spectra can be successfully simulated, as shown in Figure 8b,c, assuming *g* = 2.012 and Γ = 0.22 T for FE, *g* = 2.005 and Γ = 0.023 T for PE, and relative weights 0.09 and 0.91 for FE and PE, respectively. Hence, the two different Mn(II) centers associated with two EPR signals can be attributed to structures of **AZEMnBr** in its the FE and PE phase. However, upon cooling back to the FE phase, the EPR spectrum did not convert to its initial form, which can be attributed to the slow rebuilding of the ferroelastic domains.^64,65^

The decrease in the linewidth upon the PT can be correlated with structural changes. The XRD experiments amply proved that there is an increase in the AZE cation dynamics after the PT, and the EPR linewidths are sensitive to the fluctuations of the Mn(II) neighbours^62,69^ because such fluctuations change spin–lattice relaxation time of the paramagnetic ion. The X-ray experiment also revealed that although the [MnBr_4_]^2–^ moiety in **AZEMnBr** is not significantly altered by the PT, the arrangement of the bromide anions around Mn(II) at high temperatures is closer to the ideal tetrahedron (to cubic symmetry). Thus, the *D* parameter is expected to become closer to zero,^70^ and thus, the ZFS outer transitions do not broaden the line at its both ends.

### 3.6. Magnetic DC and AC Susceptibility

The molar magnetic susceptibility and *χ*_M_*T* (or effective magnetic moment) curves are displayed in Figure 9a, while the magnetization *vs.* magnetic field per formula unit M_1_ = *M*_mol_/*N*_A_μ_B_ at the constant temperature is shown in Figure 9b. For **AZEMnBr**, slow increase in *χ*_M_*vs*. temperature curve with the decreasing of temperature is observed which is rapid in the low-temperatures region.

The χ_M_ versus temperature curve increases slowly with the decrease in temperature, but in the low-temperature region, a rapid increase in molar susceptibility values occurs. The value of *χ*_M_*T* at room temperature is 4.44 cm^3^ mol^–1^K (5.96 *μ*_B_) similar to that expected for one Mn(II) ion without any exchange interactions (with *S* =5/2 and *g*_av_ = 2.00). This product stays constant down to *T* ∼ 30 K; then, it drops to 2.93 cm^3^ mol^–1^K (4.84 *μ*_B_) at *T* = 1.8 K (Figure 9a). The magnetic susceptibility obeys the Curie–Weiss law in the 30–300 K temperature region giving the values of *C* and Θ parameters equal to 4.44 cm^3^ mol^–1^K and −0.43 K, respectively (Figure 9a, inset). The decrease in *χ*_M_*T* at the low-temperature region can either be due to the zero-field splitting effect of the Mn(II) ions or intermolecular exchange interactions transmitted through various Mn···Mn intermolecular interactions such as hydrogen bonds characterized by the *zJ*′ parameter (where z is the number of adjacent paramagnetic species around a given mononuclear unit). To described theoretically, for the DC susceptibility and magnetization data, we used the ZFS model described by the spin Hamiltonian.
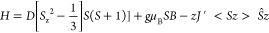
4

All data fitting has been done by exploiting PHI software.^71^

The best agreement with the experimental magnetic data for **AZEMnBr** was obtained with *zJ*′ = −0.01 cm^–1^, *g* = 2.01, *D* = 0.2 cm^–1^, and R = Σ[(χ*T*)exp – (*χT*)calc]^2^/Σ[(*χT*)exp]^2^ = 8.77 × 10^–5^. The calculated curve matches the magnetic data well.

The obtained result suggests that (i) the complex **AZEMnBr** can be treated as an almost isolate system (very low value of *zJ*′, which is consistent with the crystallographic data, short Mn···Mn distance 7.687 Å), and the intermolecular interaction transmitted through the hydrogen bond and π–π interactions is not significant and (ii) the zero-field splitting effect of the Mn^2+^ ions (*D* = 0.2 cm^–1^ agreement with EPR spectroscopy and ab initio calculations) is predominant and affects the decrease in *χ*_M_T in the low-temperature range. This effect is also well visible in magnetization versus magnetic field measurement (Figure 9b). The magnetization per formula unit *M*_1_ = *M*_mol_/(N_A_*μ*_B_) at *B* = 5 T and *T* = 2.0 K tends to saturate with the value of *M*_sat_ = 4.95 *μ*B. In such a case, the ground state equals *S* = ^5^/_2_5/2, and the magnetization should saturate to the value of 5 *μ*_B_. The obtained value under high magnetic fields is a little bit smaller and evidences the ZFS effect.

#### AC Susceptibility

AC susceptibility measurements were performed first at low temperature *T* = 2.0 K for a set of representative frequencies of the alternating field (*f* = 1.1, 11, 111, and 1111 Hz) by sweeping the magnetic field from zero to *B*_DC_ = 1 T with the working amplitude *B*_AC_ = 0.3 *μ*T. Under the zero field, no absorption signal (out-of-phase susceptibility component *χ*″) was observed due to fast magnetic tunneling.

**Figure 10:**
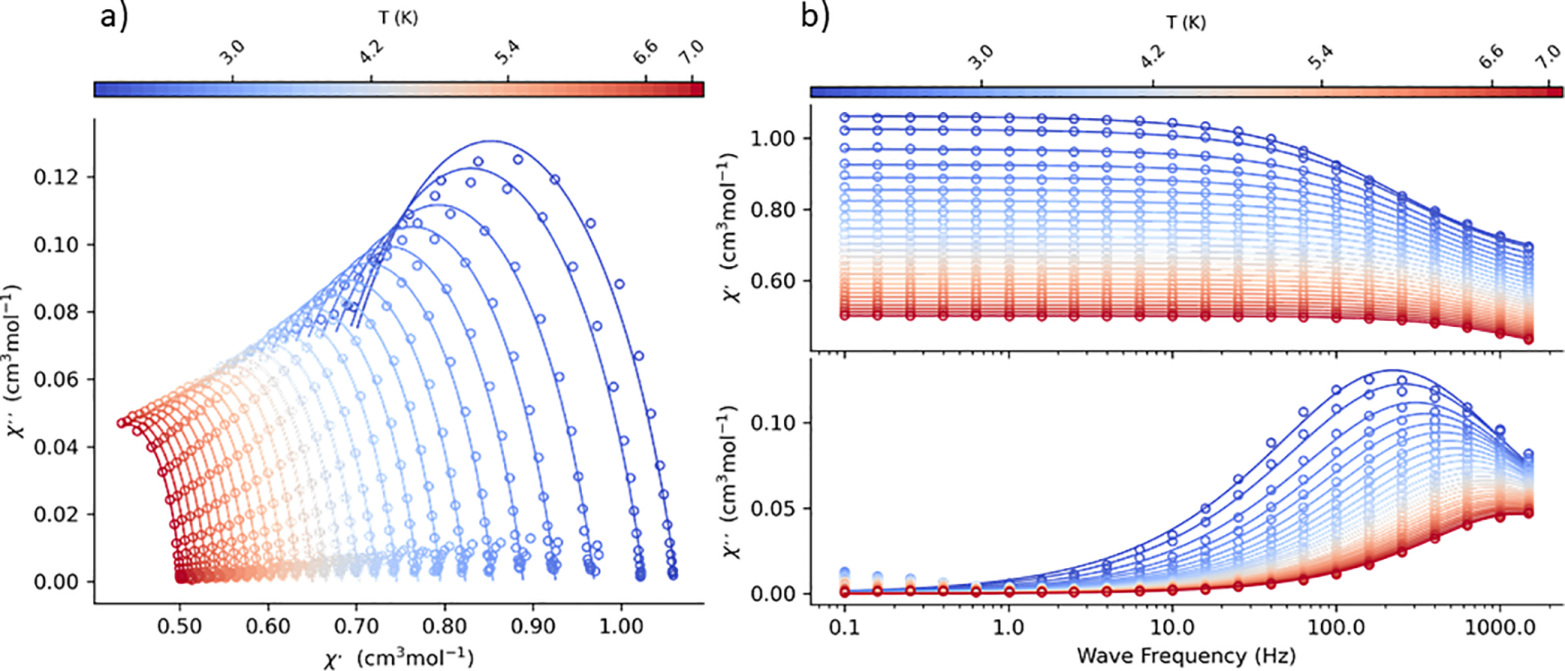
(a) Argand diagram for **AZEMnBr**. (b) Frequency dependence of the AC susceptibility components for **AZEMnBr** at *B*_DC_ = 0.1 T and fixed temperature. Lines—fitted.

With the increasing external field, this component raised and passed through a maximum between 0.3 and 0.4 T at the highest frequencies (Figure S5, Supporting Information). This behavior indicates that the crystal under study can exhibit field-induced slow magnetic relaxation. At the next step, we measured AC susceptibility under a fixed external magnetic field *B*_DC_ = 0.4 T (the maximum of the high-frequency signal), changing the frequency between *f* = 0.1 to 1500 Hz for a set of temperatures between *T* = 1.8 and 7 K (Figure S6, Supporting Information). The AC susceptibility data were fitted using CC-FIT2 software^72^ by employing the generalized Debye single relaxation time model (appropriate if the experimental Argand diagram can be recovered using a small value of the parameter α). The α parameters are in the range of 0.16 to 0.29 (Table S6, Supporting Information), suggesting a narrow distribution of relaxation times. The Argand diagram for the fixed temperature is shown in Figure 10a. The frequencies of the maxima (or fitted relaxation times) (Figure 10b) enter the Arrhenius-like plot (Figure S7, Supporting Information). It can be seen that with increasing temperature, the relaxation time is shortening as expected.

This fact allows us to determine the relaxation parameters for the Orbach process in the high-temperature limit: the barrier to spin reversal, *U*/*k*_B_ = 10 (20) K, and a relaxation time proportionality constant of τ_0_ = 10^–4(6)^ s, which is longer than the expected range of τ_0_ (10^–6^ to 10^–11^ s) for manganese compounds showing SMM.^73−76^ However, the energy barrier value is lower than that reported previously in the literature for the Mn(II) SMM complexes (typically in the range 20–60 K). The values of these parameters make it possible to unequivocally classify this compound into a group of field-induced single-ion magnet (SIM) complexes. The curved part of the Arrhenius-like plot (at the low-temperature limit) can be recovered by considering the Raman process of the relaxation using a linearized form of τ^–1^ = *CT*^*n*^. The extended relaxation equation then takes the form τ^–1^ = τ_o_^–1^ exp(*U*/*k*_B_*T*) + *CT*^*n*^ with Raman process parameters *n* = 1.2 (20) and *C* = 10^3(5)^ K^*–n*^ s^–1^.

## 4 Conclusions

A novel molecular-ionic crystal, **AZEMnBr**, has been obtained and characterized by DSC, DTA, and TGA analyses. The crystal is stable up to about 440 K. Moreover, thermal analysis indicates the existence of one structural PT at 349/346 K on heating/cooling. According to the XRD results, the low-temperature phase is monoclinic space group *P*2_1_/*n*, and in turn, the high-temperature one is orthorhombic, *Pnma*. The transition with the order–disorder mechanism was classified to the ferroelastic–paraelastic type. The X-ray analysis suggests that the anionic MnBr_4_^2–^ component is discrete in the crystal lattice and not strongly affected by the PT. However, the AZE cations exhibit distinct dynamical disorder over the high-temperature phase. The dynamical disorder is frozen below 346 K (PT temperature). This effect was used for switching between two distinct dielectric states. The absorption and luminescence measurements performed on the monocrystal show that **AZEMnBr** has excellent reversible dual-bistable (ON/OFF) photoelectric switching capability due to a reversible order–disorder PT coupled with a remarkable change in photoluminescence. The transition from the ferroelastic to paraelastic phase was also confirmed by EPR experiments, which also showed that conversion to the paraelastic phase is a slow process. The PT was observed as significant change in the EPR linewidth, which indicates that the dynamics of the AZE cation affect the spin relaxation time for the Mn^2+^ ions. The AC susceptibility data reveal that this crystal exhibits a slow magnetic relaxation under a small applied DC field (*B*_DC_ = 0.4 T) with relaxation parameters, for example, energy barrier to spin reversal and relaxation time allows this compound to be classified as a group of field-induced SIM complexes. Finally, it should be noted that the inorganic–organic hybrid **AZEMnBr** is a rare example of multifunctional materials exhibiting dielectric, magnetic, and photoluminescence activity. Combining these properties and structural flexibility, our research provides a new approach to fabricating multifunctional magneto-optoelectronic devices.

## Supporting Information Available

TGA-DTA results, crystallographic data, hydrogen bonding interaction data, computational data, and results from magnetic measurement (PDF)

## Accession Codes

CCDC 2069243–2069244 contain the supplementary crystallographic data for this paper. These data can be obtained free of charge via www.ccdc.cam.ac.uk/data_request/cif, or by emailing data_request@ccdc.cam.ac.uk, or by contacting The Cambridge Crystallographic Data Centre, 12 Union Road, Cambridge CB2 1EZ, UK; fax: +44 1223 336033.
