# Supplementary material for: Dielectric-Optical Switches: Photoluminescent, EPR, and Magnetic Studies on Organic–Inorganic Hybrid (azetidinium)2MnBr4
Source: Inorg Chem. 2022 Mar 28;61(14):5626–36. doi: 10.1021/acs.inorgchem.2c00363 (PMC9006216; doi:10.1021/acs.inorgchem.2c00363)
Supplement: Supplementary file 1 — ic2c00363_si_001.pdf [file ic2c00363_si_001.pdf]

Associated Content

Supplementary Materials

Dielectric-Optical Switches: Photoluminescent, EPR and Magnetic Studies on Organic-Inorganic Hybrid (azetidinium)$_2$MnBr$_4$

Magdalena Rok[a*], Bartosz Zarychta[b], Rafał Janicki [a], Maciej Witwicki [a], Alina Bieńko [a], Grażyna Bator [a]

* e-mail: magdalena.rok@chem.uni.wroc.pl

[a] Faculty of Chemistry, University of Wroclaw, 14 F. Joliot – Curie, 50-383 Wroclaw, Poland.

[b] Faculty of Chemistry, University of Opole, Opole PL-45052, Poland

CAPTIONS OF FIGURES

Figure S1. X-ray diffraction pattern at 298 K of **AZEMnBr** (black-dotted) and calculated from crystal structure (red-solid) along with corresponding difference plot. 3
Figure S2. The results of the simultaneous TGA/DSC analyses for **AZEMnBr** (sample mass m = 19.000mg 5K/min. 6
Figure S3. Temperature dependence of indicated emission decays. 7
Figure S4. Molecular model used in the computations along with the d-type molecular orbitals. 9
Figure S5. Field dependence of the AC susceptibility components for **AZEMnBr** at T = 2.0 K for a set of frequencies of the AC field. Lines serve as a guide for eyes. 11
Figure S6. Temperature dependence of the in-phase (a) and out-of-phase (b) molar susceptibility for **AZEMnBr**. 11
Figure S7. Arrhenius-like plot; full lines: fitted by Orbach and Raman model with Orbach process parameters $\tau = \tau_0 \exp(U/k_B T)$: U_{eff} = 10(20) K, $\tau_0 = 10^{-4(6)}$ s, and Raman process parameters, $\tau^{-1} = CT^n$: n = 0.2 (20), $C = 10^{3(5)}$ K^{-n} s^{-1}. 12

CAPTIONS OF TABLES

Table S1. X-ray experimental details for **AZEMnBr** at 200 (**II**) and 365 K (**I**). Empirical absorption correction using spherical harmonics, implemented in SCALE3 ABSPACK scaling algorithm. H-atom parameters were constrained. 2
Table S2. Selected geometric parameters (Å, º) for **AZEMnBr** at 200 (**II**) and 365 K (**I**). 4
Table S3. Selected hydrogen-bond parameters for **AZEMnBr** at 200 (**II**) and 365 K (**I**). 5
Table S4. Thermodynamic parameters of the phase transitions for **AZEMnBr** in the condensed state indicated from DSC results and calculated according to equation: $\Delta S = R\ln N^2$, $N = N_2/(N_1=1)$. 6
Table S5. Results of CASSCF/NEVPT2 calculations of the zero-field splitting parameters. The spin-orbit coupling (D^{SOC}) and spin-spin coupling (D^{SSC}) contributions to the D parameter are shown. 9
Table S6. Relaxation fitting parameters from the least-square fitting of the Cole-Cole plots of compound **AZEMnBr** according to the generalized Debye model. 13

Table S1. X-ray experimental details for **AZEMnBr** at 200 (**II**) and 365 K (**I**). Empirical absorption correction using spherical harmonics, implemented in SCALE3 ABSPACK scaling algorithm. H-atom parameters were constrained.

	(200K, phase II)	(365K, phase I)
Crystal data	**$(C_3H_8N)_2[MnBr_4]$ (1),** 490.79 g/mol	
Crystal system, space group	Monoclinic, $P2_1/n$	Orthorhombic, *Pnma*
Temperature (K)	200	365
a, *b*, *c* (Å)	8.5416 (4), 11.7663 (5), 14.7306 (7)	11.712 (6), 8.648 (5), 15.280 (8)
α, β, γ (°)	90, 93.029 (4), 90	90, 90, 90
V ($Å^3$)	1478.40 (12)	1547.7 (14)
μ (mm^{-1})	11.67	11.15
Crystal size (mm)	0.5 × 0.45 × 0.3	0.5 × 0.45 × 0.3
T_{min}, T_{max}	0.469, 1.000	0.341, 1.000
No. of measured, independent and observed [$I > 2\sigma(I)$] reflections	11559, 3744, 2344	9898, 1627, 223
R_{int}	0.033	0.242
$(\sin \theta/\lambda)_{max}$ ($Å^{-1}$)	0.689	0.617
Refinement		
$R[F^2 > 2\sigma(F^2)]$, $wR(F^2)$, S	0.028, 0.053, 0.80	0.030, 0.074, 0.51
No. of reflections	3744	1627
No. of parameters	118	100
No. of restraints	0	126
$\Delta\rho_{max}$, $\Delta\rho_{min}$ (e $Å^{-3}$)	0.59, −0.75	0.14, −0.13

Computer programs: *CrysAlis CCD* (Oxford Diffraction, 2007), *CrysAlis RED* (Oxford Diffraction, 2007), *SHELXS2013* (Sheldrick, 2013), *SHELXL2013* (Sheldrick, 2013), *SHELXTL* (Sheldrick, 2008).

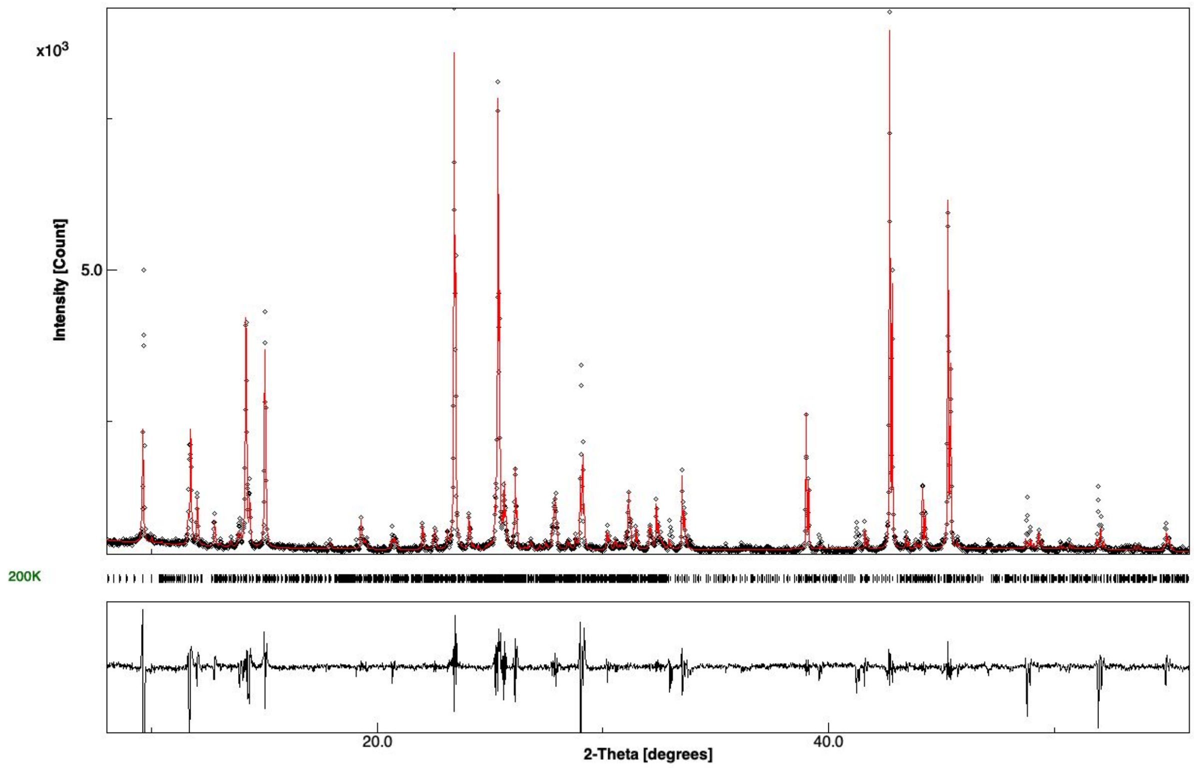

Figure S1. X-ray diffraction pattern at 298 K of **AZEMnBr** (black-dotted) and calculated from crystal structure (red-solid) along with corresponding difference plot.

Table S2. Selected geometric parameters (Å, º) for **AZEMnBr** at 200 (**II**) and 365 K (**I**).

**200 K , phase II**			
Bond distances			
Mn1—Br3	2.4762 (6)	C2—C3	1.517 (5)
Mn1—Br2	2.4954 (6)	C3—C4	1.503 (5)
Mn1—Br1	2.5075 (6)	N5—C6	1.500 (5)
Mn1—Br3'	2.5102 (6)	N5—C8	1.506 (5)
N1—C2	1.500 (4)	C6—C7	1.505 (5)
N1—C4	1.515 (4)	C7—C8	1.511 (5)
Valence angels			
Br3—Mn1—Br2	110.75 (2)	N1—C2—C3	90.0 (3)
Br3—Mn1—Br1	110.06 (2)	C4—C3—C2	89.8 (3)
Br2—Mn1—Br1	111.77 (2)	C3—C4—N1	89.9 (3)
Br3—Mn1—Br3'	113.57 (2)	C6—N5—C8	88.8 (3)
Br2—Mn1—Br3'	105.96 (2)	N5—C6—C7	89.9 (3)
Br1—Mn1—Br3'	104.56 (2)	C6—C7—C8	88.4 (3)
C2—N1—C4	90.0 (2)	N5—C8—C7	89.4 (3)
Toesion angels			
C4—N1—C2—C3	4.7 (3)	C8—N5—C6—C7	−14.2 (4)
N1—C2—C3—C4	−4.7 (3)	N5—C6—C7—C8	14.1 (3)
C2—C3—C4—N1	4.7 (3)	C6—N5—C8—C7	14.1 (4)
C2—N1—C4—C3	−4.7 (3)	C6—C7—C8—N5	−14.0 (3)
**365 K, phase I**			
Bond distances			
Mn1—Br2	2.445 (2)	C2—C3	1.45 (3)
Mn1—Br3^{i}	2.4615 (17)	C3—C4	1.22 (3)
Mn1—Br3	2.4615 (17)	N5—C6	1.30 (2)
Mn1—Br1	2.465 (3)	N5—C8	1.62 (2)
N1—C2	1.17 (3)	C6—C7	1.56 (3)
N1—C4	1.70 (3)	C7—C8	1.18 (2)
Valance angles			
Br2—Mn1—Br3^{i}	109.94 (6)	N1—C2—C3	97 (4)
Br2—Mn1—Br3	109.94 (6)	C4—C3—C2	95 (4)
Br3^{i}—Mn1—Br3	110.67 (9)	C3—C4—N1	83 (3)
Br2—Mn1—Br1	112.94 (8)	C6—N5—C8	95 (2)
Br3^{i}—Mn1—Br1	106.63 (6)	C8—C7—C6	103 (3)
Br3—Mn1—Br1	106.63 (6)	C7—C8—N5	82 (2)
C2—N1—C4	84 (3)		
Torsion angles			
C4—N1—C2—C3	5 (3)	N5—C6—C7—C8	4 (3)
N1—C2—C3—C4	−7 (4)	C6—N5—C7—C8	−175 (3)
C2—C3—C4—N1	5 (3)	C8—N5—C7—C6	175 (3)
C2—N1—C4—C3	−6 (3)	C6—C7—C8—N5	−4 (2)
C8—N5—C6—C7	−3 (2)	C6—N5—C8—C7	4 (3)

Symmetry code(s): (i) x, $-y+3/2$, z

Table S3. Selected hydrogen-bond parameters for **AZEMnBr** at 200 (**II**) and 365 K (**I**).

D—H···*A*	*D*—H (Å)	H···*A* (Å)	*D*···*A* (Å)	*D*—H···*A* (°)
**200 K, phase II**				

N1—H1A···Br1^{i}	0.97	2.46	3.365 (3)	155.6
N1—H1B···Br2^{ii}	0.97	2.91	3.500 (3)	120.3
N1—H1B···Br3'^{ii}	0.97	2.58	3.408 (3)	142.9
N5—H5A···Br3^{ii}	0.97	2.41	3.374 (3)	172.2
N5—H5B···Br1^{iii}	0.97	2.94	3.472 (3)	116.0
N5—H5B···Br3'^{iii}	0.97	2.61	3.440 (3)	143.8
**365 K, phase I**				
N1—H1A···Br1^{iv}	0.90	2.95	3.61 (2)	131.9
N1—H1B···Br2^{v}	0.90	2.95	3.83 (3)	166.1
C2—H2B···Br3	0.96	2.95	3.44 (3)	113.2
C3—H3A···Br3^{vi}	0.96	2.73	3.49 (3)	136.3
C4—H4B···Br2^{iv}	0.96	2.86	3.44 (2)	119.9
N5—H5A···Br1^{vii}	0.90	3.05	3.78 (2)	139.6
C7—H7A···Br3	0.96	3.06	3.58 (3)	116.2
C8—H8A···Br3^{v}	0.96	3.13	3.76 (2)	124.7

Symmetry code(s): (i) $x-1/2, -y+1/2, z-1/2$; (ii) $-x+3/2, y+1/2, -z+1/2$; (iii) $-x+2, -y+1, -z+1$; (iv) $-x+1/2, -y+1, z+1/2$; (v) $x+1/2, y, -z+3/2$; (vi) $x+1/2, -y+1/2, -z+3/2$; (vii) $-x+1, -y+2, -z+1$.

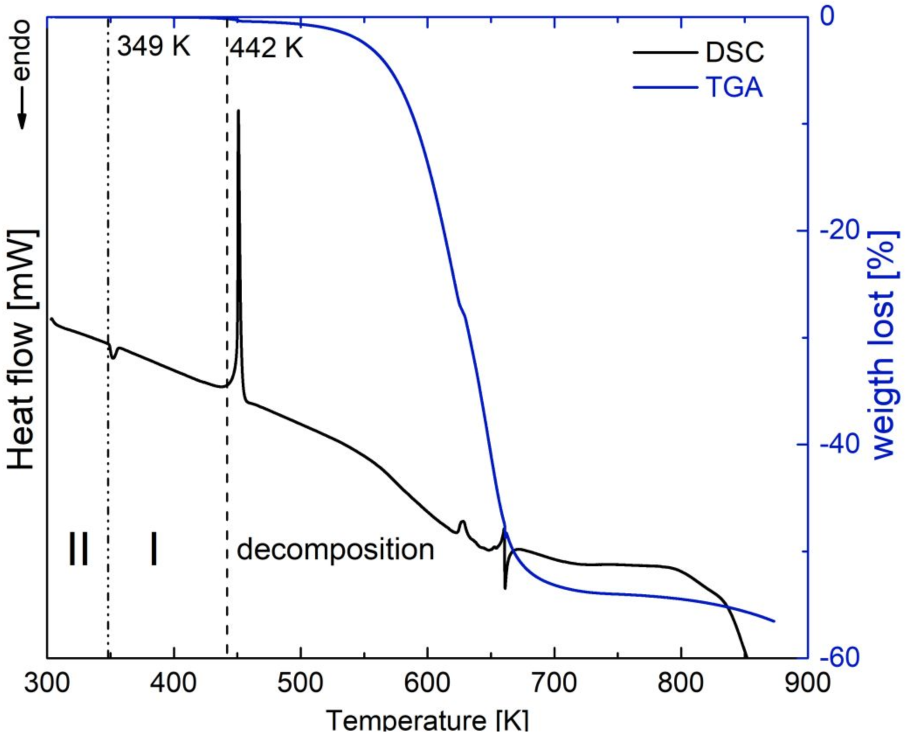

Figure S2. The results of the simultaneous TGA/DSC analyses for **AZEMnBr** (sample mass m = 19.000mg 5K/min.

Table S4. Thermodynamic parameters of the phase transitions for **AZEMnBr** in the condensed state indicated from DSC results and calculated according to equation: $\Delta S = R\ln N^2$, $N = N_2/(N_1=1)$.

**PT**	**II→I**
**Compound**	$(C_3H_8N)_2[MnBr_4]$ (**1**)
**M [g/mol]**	490.8
**T [K]**	346.2
**ΔH [J·g^{-1}]**	24.4
**ΔH [J·mol^{-1}]**	11975.3
**ΔS [J·mol^{-1}·K^{-1}]**	34.6
N	8.0

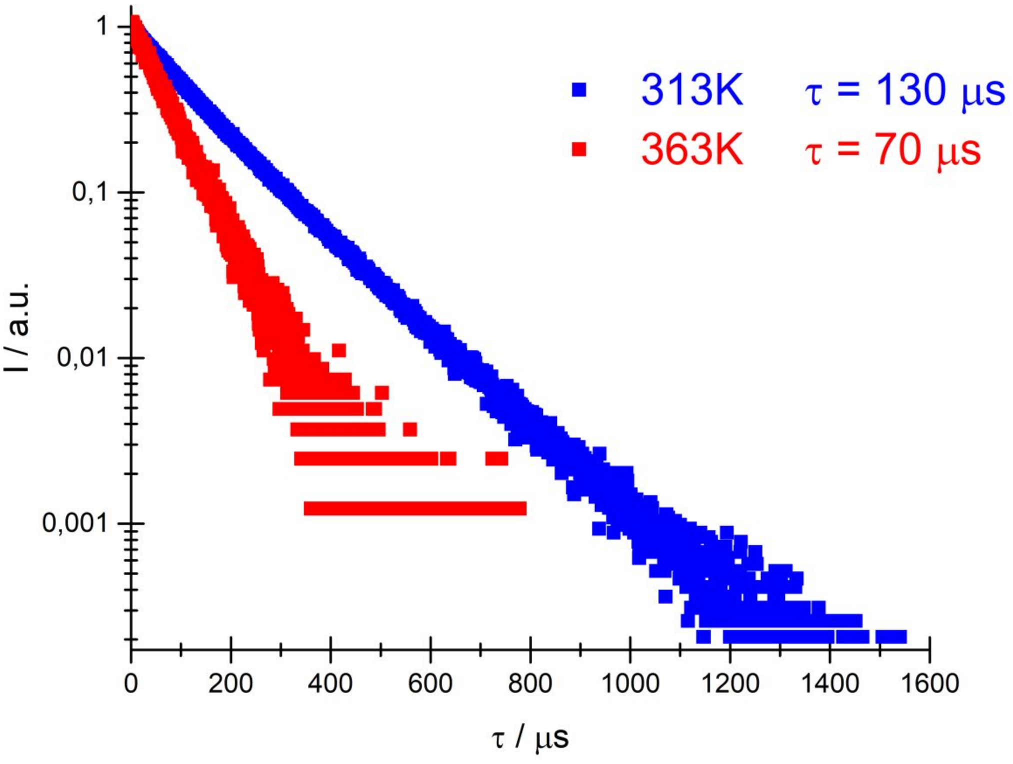

Figure S3. Temperature dependence of indicated emission decays.

Analysis of the D parameter

To confirm this magnitude of D, we carried out the CASSCF/NEVPT2 computations. Figure S3 (ESI) shows the molecular model ($[AZE]_2[MnBr_4]$) used in the computations, and Table S5 (ESI) summarises their results. The calculation of one sextet (the ground state) and 24 quartet states with the active space consisting of five electrons in the five 3d Mn(II) orbitals, i.e. CAS(5,5), produced the parameter D = +0.020 cm^{-1}, that is tenfold lower than that in the SQUID experiment. The inclusion of 75 doublet states in the CAS(5,5) computations had a minimal effect on the calculated D parameter value (0.023 cm^{-1}). However, the CAS(5,5) active space does not explicitly include the correlation of the bromine electrons. For complexes containing heavy halogens, the inclusion of halogens' p-electrons in the active space plays a crucial role in the description of the electronic structure.[1,2] Hence, we extended the active space to 29 electrons in 17 orbitals (the inclusion of all 4p orbitals of the bromines), that is CAS(29,17), and performed calculations for one sextet, 24 quartet and 75 doublet states. The predicted D parameter value slightly improved (+0.027 cm^{-1}), but remained underestimated compared to the experiment. This suggested that ligand-to-metal charge transfer states can have a significant effect on the D parameter value. Although Mn(II) shows limited covalent bonding to the bromine ligands (Figure S3, ESI) and thus the ligand-to-metal charge transfer states are expected to be high in energy, their spin-orbit coupling with the ground state can be important because of relatively strong spin-orbit effects for heavy atoms like bromine.

The CASSCF/NEVPT2 computations with CAS(29,17) for 37 sextets (36 excited sextets), 24 quartet and 75 doublet states indeed showed that the first excited sextet state was about 53200 cm^{-1} above the ground state. In contrast, the first quartet and doublet states were separated from the ground state by 23900 and 35000 cm^{-1}, respectively The inclusion of the excited sextet states significantly improves the predicted value of D (+0.11 cm^{-1}), but the predicted D parameter remains underestimated. This is understandable because the number of excited sextet states in the calculations was limited. Moreover, the further improvement of the D prediction possibly requires the inclusion of the bromines' 4s and unoccupied (virtual) orbitals in the active space. However, such extended calculations at the CASSCF/NEVPT2 theory level become infeasible. All in all, the performed CASSCF/NEVPT2 computations confirmed that the D parameter value for **AZEMnBr** is small and its sign is positive.

(1) Mishra, S. Structural and Spectroscopic Study of the Excited Electronic States of Silver Dihalides by Quantum Chemical Methods. *Phys. Chem. Chem. Phys.* **2008**, *10* (27), 3987–3991.

(2) Chowdhury, S. R.; Mishra, S. Heavy Ligand Atom Induced Large Magnetic Anisotropy in Mn(Ii) Complexes. *Phys. Chem. Chem. Phys.* **2017**, *19* (25), 16914–16922.

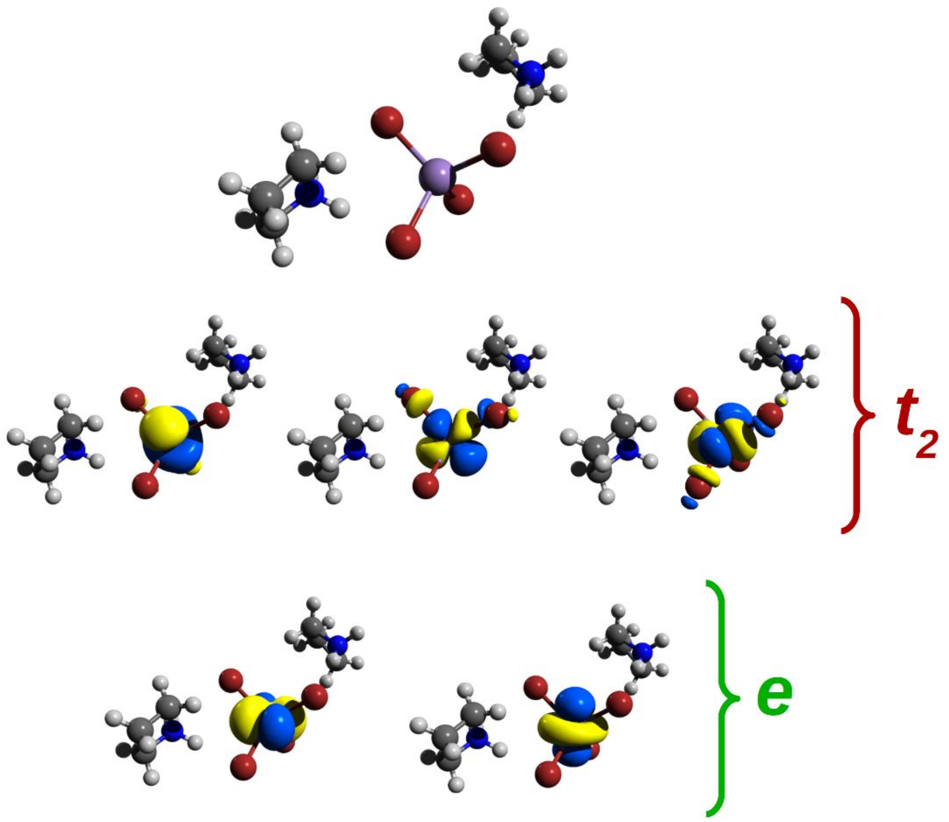

Figure S4. Molecular model used in the computations along with the d-type molecular orbitals.

Table S5. Results of CASSCF/NEVPT2 calculations of the zero-field splitting parameters. The spin-orbit coupling (D^{SOC}) and spin-spin coupling (D^{SSC}) contributions to the D parameter are shown.

	number of electronic states						
	sextet	quartet	doublet	D [cm^{-1}]	D^{SOC} [cm^{-1}]	D^{SSC} [cm^{-1}]	E [cm^{-1}]
CAS(5,5)	1	24	0	0.020	0.016	0.004	0.003
	1	24	75	0.023	0.018	0.005	0.003
CAS(29,17)	1	24	0	0.025	0.020	0.005	0.005
	1	24	75	0.027	0.022	0.005	0.005
	37	24	75	0.108	0.103	0.005	0.005

Analysis of the g parameter

Another important aspect of EPR spectra of **AZEMnBr** at 200 K is that the g value (g = 2.014) is considerably larger than reported g = 2.003 and g = 2.008 for $[Mn(CH_3CN)_6]^{2+}$ and $[MnBr_4]^{2-}$ ions, respectively, but comparable to the methyltriphenylphosphonium salts of $[MnBr_4]^{2-}$ and $[MnCl_2Br_2]^{2-}$ exposed to isoamyl alcohol vapours (g = 2.016 and g = 2.012, respectively).[1,2] In contrast to D, the g parameter value is affected only by the spin–orbit coupling of the excited states that conserve the spin of the ground state.[3,4] For high-spin Mn(II) complexes, the d-d excitations cannot conserve the total spin. Hence, the elevated value of g for **AZEMnBr** results from the bromine-to-Mn(II) charge transfer states, which we have also shown as important for the D parameter. The g_{avg} value, calculated as $(g_x+g_y+g_z)/3$, obtained from the CASSCF/NEVPT2 computations with CAS(29,17) for 37 sextets, 24 quartet and 75 doublet states amounts to 2.006. This underestimation shows that likewise for the D parameter, for the CASSCF/NEVPT2 computations, a larger active space and the larger number of sextet states are required for an improved agreement with the experiment. A quantitatively better prediction of the g parameter was performed at the DFT B3LYP (g_{avg} = 2.012) and PBE0 (g_{avg} = 2.010) theory level because the DFT-based approach includes contributions from all sextet states.

(1) Chan, S. I.; Fung, B. M.; Lütje, H. Electron Paramagnetic Resonance of Mn(II) Complexes in Acetonitrile. J. Chem. Phys. 1967, 47 (6), 2121–2130.

(2) Balsamy, S.; Natarajan, P.; Vedalakshmi, R.; Muralidharan, S. Triboluminescence and Vapor-Induced Phase Transitions in the Solids of Methyltriphenylphosphonium Tetrahalomanganate(II) Complexes. Inorg. Chem. 2014, 53 (12), 6054–6059.

(3) Neese, F. Importance of Direct Spin-Spin Coupling and Spin-Flip Excitations for the Zero-Field Splittings of Transition Metal Complexes: A Case Study. J. Am. Chem. Soc. 2006, 128 (31), 10213–10222.

(4) Boudreaux, E. A.; Mulay, L. N. Theory and Applications of Molecular Paramagnetism; Wiley-Interscience: New York, 1976.

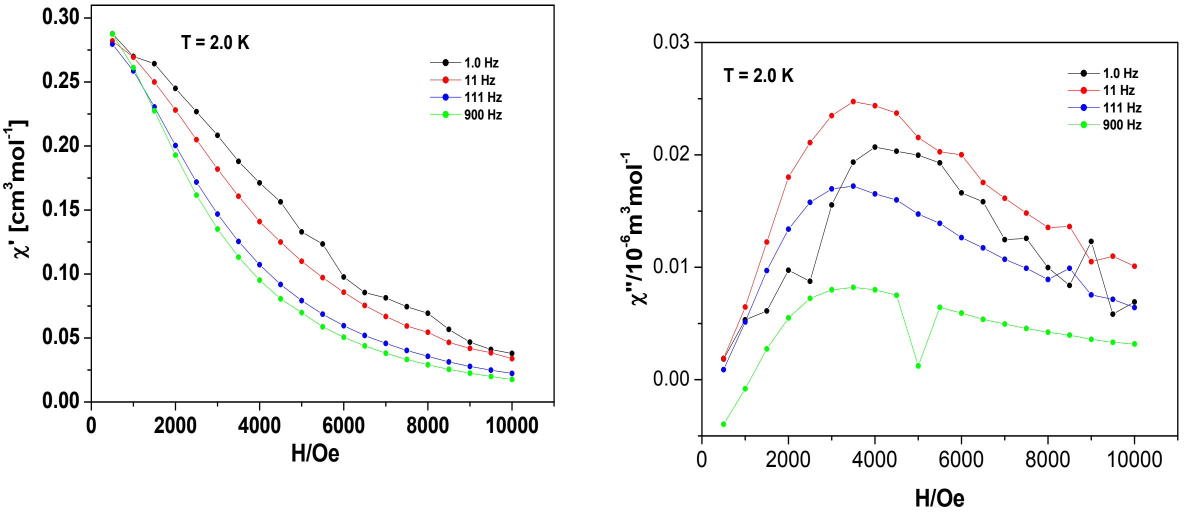

Figure S5. Field dependence of the AC susceptibility components for **AZEMnBr** at T = 2.0 K for a set of frequencies of the AC field. Lines serve as a guide for eyes.

a) b)

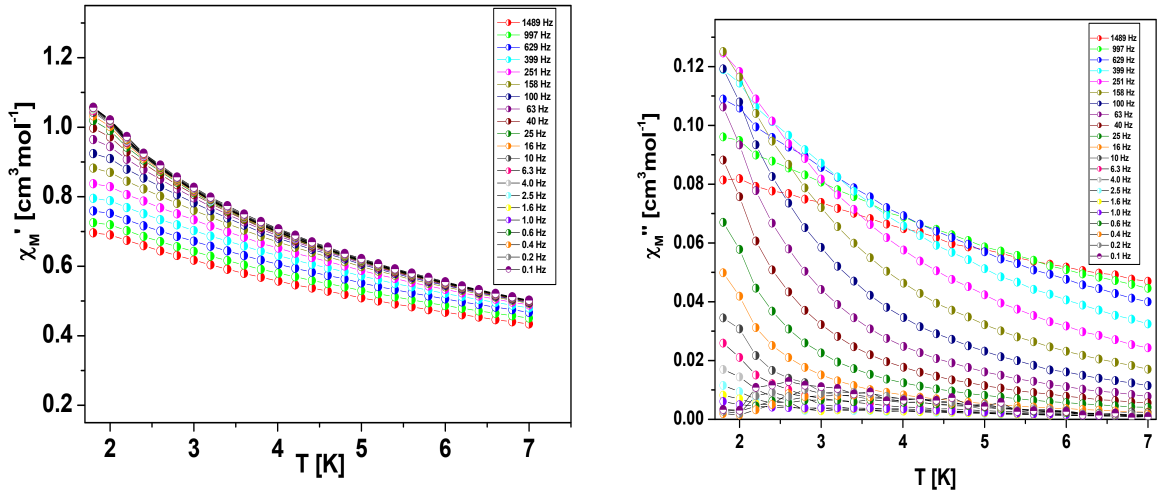

Figure S6. Temperature dependence of the in-phase (a) and out-of-phase (b) molar susceptibility for **AZEMnBr**.

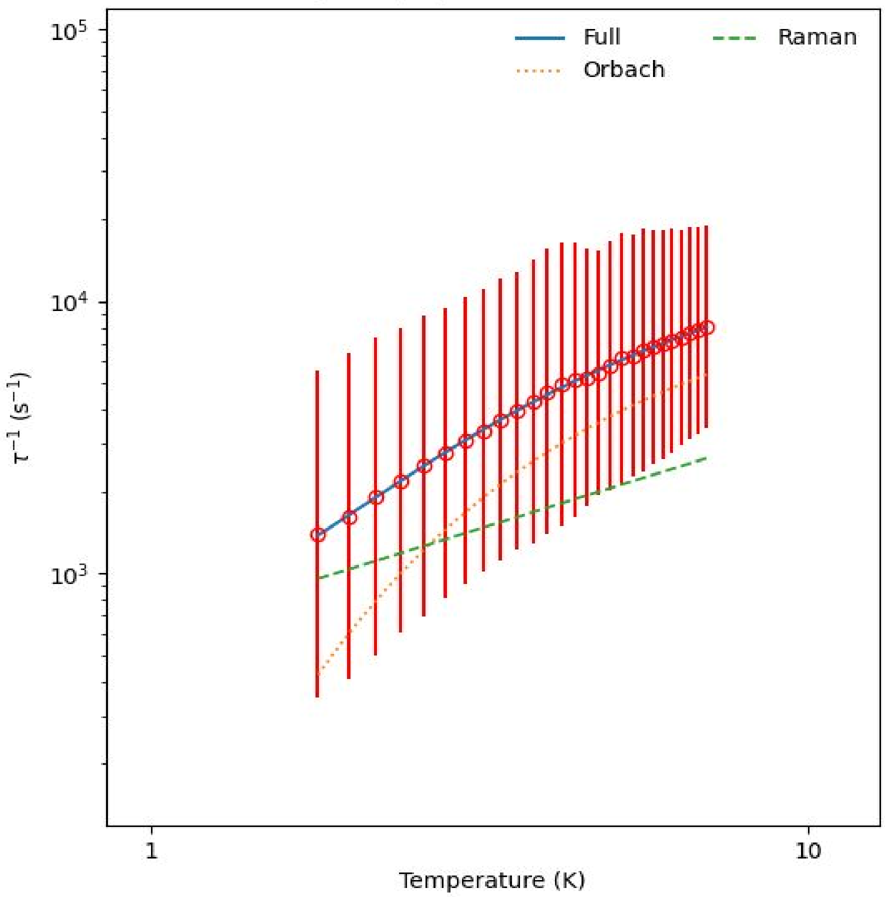

Figure S7. Arrhenius-like plot; full lines: fitted by Orbach and Raman model with Orbach process parameters $\tau = \tau_0 \exp(U/k_B T)$: U_{eff} = 10(20) K, $\tau_0 = 10^{-4(6)}$ s, and Raman process parameters, $\tau^{-1} = CT^n$: n = 0.2 (20), $C = 10^{3(5)}$ K^{-n} s^{-1}.

Table S6. Relaxation fitting parameters from the least-square fitting of the Cole-Cole plots of compound **AZEMnBr** according to the generalized Debye model.

T/K	τ /s	χ_S [cm^{3}mol^{-1}K]	χ_T[cm^{3}mol^{-1}K]	α
1,79	0,0007168	0,642	1,062	0,29
2,00	0,0006180	0,633	1,025	0,29
2,20	0,0005222	0,616	0,969	0,28
2,40	0,0004565	0,601	0,925	0,27
2,60	0,0004028	0,584	0,889	0,26
2,80	0,0003615	0,570	0,854	0,25
3,00	0,0003242	0,556	0,823	0,25
3,20	0,0002991	0,543	0,795	0,24
3,40	0,0002723	0,529	0,769	0,24
3,60	0,0002527	0,517	0,745	0,24
3,81	0,0002341	0,504	0,723	0,25
4,01	0,0002144	0,491	0,703	0,25
4,21	0,0002025	0,481	0,684	0,25
4,40	0,0001931	0,473	0,666	0,24
4,60	0,0001914	0,469	0,650	0,22
4,80	0,0001835	0,461	0,633	0,20
5,00	0,0001727	0,450	0,618	0,21
5,20	0,0001625	0,440	0,603	0,21
5,40	0,0001589	0,433	0,589	0,20
5,60	0,0001509	0,424	0,577	0,20
5,80	0,0001480	0,419	0,564	0,19
6,00	0,0001437	0,412	0,553	0,19
6,20	0,0001391	0,405	0,541	0,18
6,40	0,0001358	0,400	0,530	0,17
6,60	0,0001306	0,392	0,520	0,17
6,80	0,0001276	0,387	0,510	0,16
7,00	0,0001235	0,381	0,501	0,16
